# Only the ‘tip of the iceberg’

**DOI:** 10.1080/2090598X.2019.1589752

**Published:** 2019-04-18

**Authors:** Gamal M. Ghoniem, Kathleen C. Kobashi

**Affiliations:** Pelvic Reconstructive Surgery and Voiding Dysfunction, Department of Urology, University of California (UC) Irvine Health, Orange, CA, USA; Section of Urology and Renal Transplantation, Virginia Mason Medical Center, Seattle, WA, USA


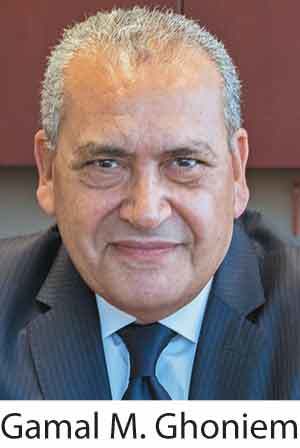

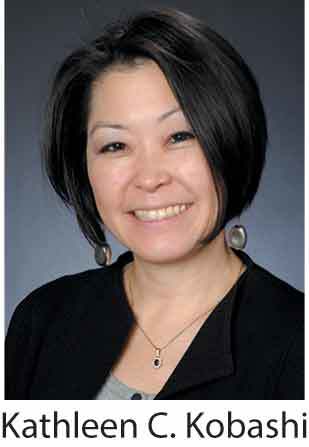
The growth of Female Pelvic Medicine and Reconstructive Surgery (FPMRS) in the Middle East has been inarguably palpable. This progress is evidenced by the dedication of numerous sessions in recent Egyptian Urological Association and Arab Urological meetings to FPMRS, as well as the recognition of prominent centres in the region that focus on women’s health issues and female urology in particular. This phenomenon likely represents only the ‘tip of the iceberg’, as the advancement of FPMRS has gained tremendous momentum and attention as of late. One possible explanation for this trend is that the prevalence of pelvic organ prolapse (POP) is estimated to be as high as 8%. In the USA, ~300 000 women per annum undergo surgical procedures for correction of POP [1]. Additionally, POP represents only a fraction of patients with pelvic floor disorders, as functional concerns can occur independent of anatomical abnormalities.

In his previously published editorial, ‘The Elephant in the Room’ Ghoniem [2] emphasised the need for collaboration between medical disciplines that interface at the subspecialty of FPMRS in order to maximise our collective knowledge and hone our skills as clinicians and surgeons. Women with POP beyond the hymen often experience concomitant urinary symptoms such as urinary incontinence; difficulty voiding, or urinary urgency; and defaecatory symptoms, such as stool trapping or faecal incontinence that may or may not be related to the POP [3].

The question is; what is being done about this pervasive quality of life-affecting problem? The answer is whilst there are ongoing efforts to better understand and address these impactful issues, there remains a lot of work to be done. Even though the situation is improving around the globe, a shortage of structured educational programmes and FPMRS fellowships continues, and an unfortunate deficit in effective collaboration between subspecialties that share interfaces in FPMRS persists.

But just as it is not possible for one to stand on a beach and prevent the tide from rolling in, physicians cannot ignore the growing social demand to develop specialised, patient-centric care for patients with pelvic floor dysfunction. The see-no-evil approach cannot persist in the face of an ageing, yet as a group, largely active, population of women who desire to continue their active lifestyles.

In this special issue of the *Arab Journal of Urology*, FPMRS experts from around the world have come together to discuss a broad spectrum of pelvic floor issues, covering topics that range from clinical to basic science foci and office-based to surgical approaches. The extensive coverage of this issue notwithstanding, it is by no means exhaustive as FPMRS is evolving and much work awaits us in the development of this exciting discipline. The good news is that opportunities abound for pelvic floor surgeons interested in advancing FPMRS. Collectively, we have so much to learn and discover, and incredible innovation is undeniably on the horizon. Ultimately, the work presented in this special issue and the results of labours yet to come to fruition will no doubt lead to improved care of our patients. Our continued endeavours will allow women with pelvic floor disorders to regain pelvic floor health and subsequently reclaim their ability to live life with quality and dignity.
